# A conceptual framework to assess effectiveness in wheelchair provision

**DOI:** 10.4102/ajod.v6i0.355

**Published:** 2017-09-08

**Authors:** Deepan C. Kamaraj, Nathan Bray, Karen Rispin, Padmaja Kankipati, Jonathan Pearlman, Johan Borg

**Affiliations:** 1 Human Engineering Research Laboratories, VA Pittsburgh Healthcare System, United States; 2 Department of Rehabilitation Science & Technology, School of Health and Rehabilitation Sciences, University of Pittsburgh, United States; 3 Centre for Health Economics and Medicines Evaluation, School of Healthcare Sciences, Bangor University, United Kingdom; 4 Department of Biology, LeTourneau University, United States; 5 Specialized Mobility Operations and Innovation Pvt. Ltd, India; 6 Social Media and Global Health, Lund University, Sweden

## Abstract

**Background:**

Currently, inadequate wheelchair provision has forced many people with disabilities to be trapped in a cycle of poverty and deprivation, limiting their ability to access education, work and social facilities. This issue is in part because of the lack of collaboration among various stakeholders who need to work together to design, manufacture and deliver such assistive mobility devices. This in turn has led to inadequate evidence about intervention effectiveness, disability prevalence and subsequent costeffectiveness that would help facilitate appropriate provision and support for people with disabilities.

**Objectives:**

In this paper, we describe a novel conceptual framework that can be tested across the globe to study and evaluate the effectiveness of wheelchair provision.

**Method:**

The Comparative Effectiveness Research Subcommittee (CER-SC), consisting of the authors of this article, housed within the Evidence-Based Practice Working Group (EBP-WG) of the International Society of Wheelchair Professionals (ISWP), conducted a scoping review of scientific literature and standard practices used during wheelchair service provision. The literature review was followed by a series of discussion groups.

**Results:**

The three iterations of the conceptual framework are described in this manuscript.

**Conclusion:**

We believe that adoption of this conceptual framework could have broad applications in wheelchair provision globally to develop evidence-based practices. Such a perspective will help in the comparison of different strategies employed in wheelchair provision and further improve clinical guidelines. Further work is being conducted to test the efficacy of this conceptual framework to evaluate effectiveness of wheelchair service provision in various settings across the globe.

## Introduction

Wheelchairs are key assistive products that help improve the quality of life of people with disabilities (Shore & Juillerat 2012; World Health Organization 2016). Their use encourages community participation (Mortenson et al. 2012; Salminen et al. 2009), increases access to education (Dudgeon, Massagli & Ross 1997) and provides better opportunities of employment (Borg et al. 2012) for people with disabilities. These life-changing products could decrease healthcare expenditures and influence national and global economies (Bray et al. 2014; Greer, Brasure & Wilt 2012a). The World Health Organization (WHO) estimates that between 10% (WHO 2008a) and 15% (WHO 2011) of the world’s population (about one billion people) and 5% of children worldwide (around 95 million children aged 14 or under) have a disability (WHO 2008b). One in ten people with a disability requires a wheelchair for their mobility (Sheldon 2006). Estimates indicate that over 20 million people who need wheelchairs for their everyday mobility are unable to obtain them (WHO 2008a, 2008c, 2011).

Independent mobility is a human right: Signatory countries to the Convention on the Rights of Persons with Disabilities are mandated to ensure that their citizens can equitably access affordable assistive products, including wheelchairs to promote mobility and independence (Borg et al. 2009; UN 2007). Currently, absent or inadequate wheelchair provision has forced many people with disabilities into a cycle of poverty and deprivation, limiting their access to education, work and social facilities (Borg et al. 2009; WHO 2008b). This lack of access is in part because of the lack of collaborations among various stakeholders who need to work together to design, manufacture and deliver wheelchairs. In turn, this has led to inadequate evidence for intervention effectiveness, disability prevalence and subsequent costeffectiveness that would help facilitate appropriate provision and support for people with disabilities (WHO 2011). The WHO guidelines on the provision of manual wheelchairs in less-resourced settings provide a broad overview of the functions of wheelchair services (WHO 2008c). These comprehensive guidelines outline eight key sequential steps involved in the wheelchair service delivery process. Independent researchers have also conducted qualitative studies to identify stakeholders and their contributions that would have to be addressed during wheelchair provision (Batavia, Batavia & Friedman 2001; Eggers et al. 2009; Greer, Brasure & Wilt 2012b). However, quantitatively evaluating stakeholder contributions and strategies adopted to accomplish the eight steps in various settings in different parts of the world is still a challenge. Developing a process to study and understand the relationships between the various stakeholders and their contributions will aid in comparing the effectiveness of various strategies adopted to accomplish these eight steps. Stringent methodologies utilising the core principles of Comparative Effectiveness Research (Brophy 2015; Brouwers et al. 2012; Dahabreh et al. 2008) could help employ analytical techniques used in health economics to facilitate more cost-effective and efficient provision of wheelchairs. The aim of this pilot work is to establish a common conceptual framework that can be tested across the globe to study and evaluate the effectiveness of wheelchair provision.

## Conceptual framework and its applications

The Comparative Effectiveness Research Subcommittee (CER-SC), consisting of the authors of this article, is housed within the Evidence-Based Practice Working Group (EBP-WG) of the International Society of Wheelchair Professionals (ISWP). The EBP-WG aims to identify and assess opportunities to improve the adoption of best practices in the field of wheelchair provision through coordination with stakeholders on a global scale. The CER-SC comprises wheelchair professionals and researchers from across the globe, and acts as the core support group for studies that evaluate effectiveness of wheelchair provision. The expertise of the CER-SC spans a wide range of disciplines, including biology, rehabilitative engineering, health economics, social medicine and global health. The CER-SC used its own familiarity with the literature and undertook a scoping literature search (using Google Scholar, PubMed and PsycInfo) to implement a two-step process in the development of the conceptual framework. The first step defined effectiveness as it pertains to wheelchair provision and the second step involved the development of the conceptual framework to evaluate the effectiveness of wheelchair provision. The framework was primarily developed through discussions within the CER-SC, with continuous mapping of concepts and terminology. This was an iterative process drawing on the expertise of each member of the CER-SC. Where there was disagreement, further discussion and literature searching took place to reach consensus.

**FIGURE 1:**
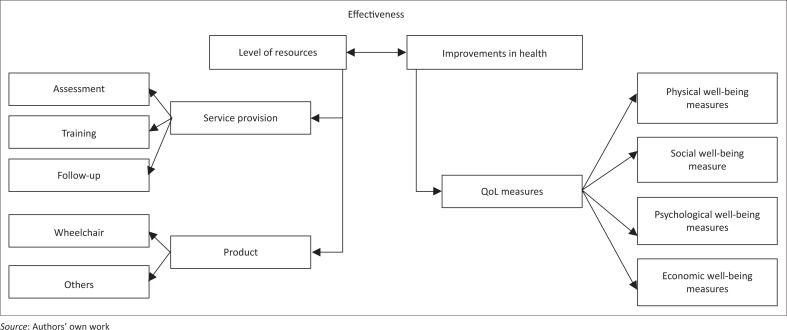
Version 1 of the conceptual framework.

In order to guide the conceptualisation process and establish common terminologies for further discussion, *wheelchair provision* is defined as an overarching term used to describe the process of wheelchair design, production, supply and service delivery (WHO 2008c); *effectiveness* is defined as the relationship between the level of resources invested in wheelchair provision and the level of results, or improvements in health (Branch & Madore 1993). Further, assessing effectiveness compares two things that have the same effect or the same purpose. The economic dimension of effectiveness alludes to cost, encompassing the concepts of cost-effectiveness and cost reduction. Thus, effectiveness can be studied in terms of clinical and economic aspects of healthcare (Branch & Madore 1993). Based on these definitions, Version 1 of the framework (Figure 1) was developed to identify various stakeholders in wheelchair provision and potential outcome variables that can be studied to evaluate effectiveness.

**FIGURE 2:**
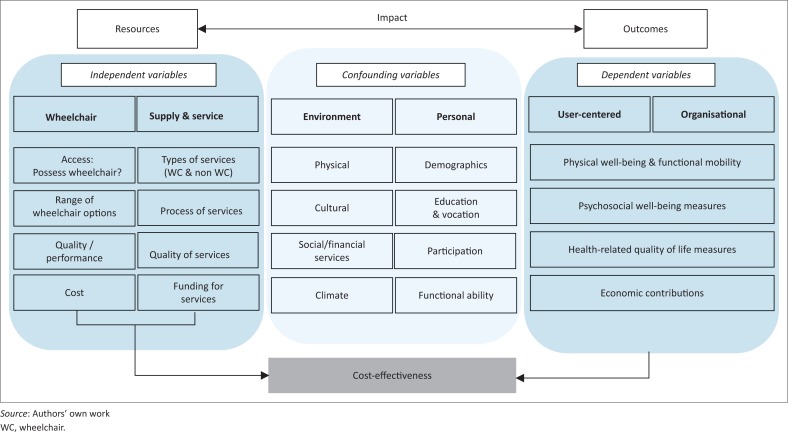
Conceptual framework illustrating the factors that affect wheelchair provision.

Further discussions led to the second version of the framework, which included the eight steps from the WHO guidelines for provision of manual wheelchairs in less-resourced settings (WHO 2008c), to provide a detailed description of the processes of wheelchair service provision. The structure of the framework was revised to illustrate the interdependency between the various factors of the process. The third and the final iteration (Figure 2) included domains from the International Classification of Functioning and Disability (WHO 2001) and the wheelchair service provision guidelines from the Rehabilitation Engineering Society of North America (Arledge et al. 2011) to include two key domains: environmental and personal factors that play an indirect role in the wheelchair service provision process. To maintain a simple and pragmatic framework that could be adopted for everyday clinical practice and effectiveness evaluation, key factors involved in wheelchair provision were categorised into three groups of variables with domains and subdomains within each group. The underlying concept echoes the idea that a group of *independent variables* interacting with each other impact the users’ everyday life. This impact can be studied using a group of *dependent variables*, while accounting for certain *confounding variables* that affect the relationship between the dependent and independent variables.

Factors pertaining to the wheelchair constituted the first domain of the independent variables. The factors pertaining to the wheelchair and the eight steps from WHO were discussed under two domains of independent variables. Access to a suitable wheelchair and various aspects of wheelchair design and production (such as types of wheelchairs, meeting ISO standards, durability, reparability and availability of spare parts that directly impact the life of a wheelchair) were included in the first domain. Factors pertaining to wheelchair supply and service delivery (types or models of services, processes, their quality and cost) constituted the second domain of the independent variables. The impact of the interaction between the two domains of the independent variables could be studied using a set of outcome variables, defined as dependent variables. Depending on the focus of these outcome variables, they were classified into two domains: administrative or organisational factors at the community level and factors pertaining to the wheelchair users at an individual level. Within each domain, the impact of the wheelchair provision on the individual’s or the community’s well-being can be evaluated under four key subdomains: physical well-being and functional mobility, psychosocial well-being, health-related quality of life and economic contributions.

The relationship between the independent and dependent variables, that is, the relationship between the wheelchair provision and the outcomes, could be affected by environmental and personal factors, contributing to the two domains of the confounding variables. The built environments, geographic locations, climate conditions along with the social and financial infrastructures constitute the environmental subdomain. Demographic factors like ethnicity, religion, functional ability, education, vocation and participation constitute the personal subdomain. To specifically illustrate the concept of cost-effectiveness and define it within the realm of wheelchair provision, it is identified as a separate domain defined by the relationship between the costs of wheelchair provision and the impact or the outcome it produces as measured by the dependent variables.

The relationships between the independent and confounding variables have a profound effect on the effectiveness of wheelchair provision. For example, if we are to compare the psychosocial impact (user-related outcomes subdomain) of two models of wheelchair provision, we must consider not only the wheelchair user’s clinical needs, but also the environment where the device would be used (environmental domain), level of wheelchair skill (personal domain) and the quality of wheelchair equipment available (wheelchair domain).

Further work is being conducted to test the efficacy of this conceptual framework to evaluate effectiveness of wheelchair service provision in various settings across the globe. A repository of outcome measures is being developed to quantify these factors, and establish the relationships between the various domains and subdomains. Future work will be focused on developing newer measurement tools, adopting psychometric and econometric methods to analyse and study the relationships of these various factors using the identified outcome measures.

In 2016, the United Nations established 17 Sustainable Development Goals (SDGs) to combat poverty, reduce inequality, protect the environment and ensure all people can live in safety. As noted by Tebbutt et al. (2016), assistive products are an ‘essential component for inclusive sustainable development’ (Tebbutt et al. 2016). For these SDGs to be realised, the provision of adequate assistive products such as wheelchairs must become a priority for governments. The wider economic, social and environmental benefits of appropriate provision of assistive technology should not be underestimated. However, universal provision of such devices cannot be achieved in an efficient and cost-effective manner unless evidence-based practices are established to guide such provision.

We believe that adoption of this conceptual framework could have broad applications in wheelchair provision globally to develop evidence-based practices. Such a perspective will help in the comparison of different strategies employed in wheelchair provision and further improve clinical guidelines. Further, this methodical approach will lay the groundwork to evaluate the efficiency and effectiveness of wheelchair provision, which can be used to advocate for the rights of people with disabilities and to draft informed policies aimed at promoting participation of people with disabilities.

## Conclusion

Wheelchair provision is a complex rehabilitation intervention necessitating cooperation and collaboration between various healthcare or rehabilitation professionals and wheelchair users. However, studying the effectiveness of wheelchair service delivery models and the relationship between the various stakeholders has been a hurdle faced by rehabilitation professionals. To address this gap in scientific literature, this project adopted the principles of Comparative Effectiveness Research to develop a cohesive conceptual framework that could be used to evaluate wheelchair provision. Through the development of this framework, we hope to provide researchers, clinicians and policymakers a structured approach with common terminologies to identify key variables from the myriad factors that need to be considered when evaluating the effectiveness of wheelchair provision. Future work will aim to evaluate the efficacy of this framework.
